# Fluorescent Proteins, Promoters, and Selectable Markers for Applications in the Lyme Disease Spirochete Borrelia burgdorferi

**DOI:** 10.1128/AEM.01824-18

**Published:** 2018-11-30

**Authors:** Constantin N. Takacs, Zachary A. Kloos, Molly Scott, Patricia A. Rosa, Christine Jacobs-Wagner

**Affiliations:** a Microbial Sciences Institute, Yale West Campus, West Haven, Connecticut, USA; b Department of Molecular, Cellular, and Developmental Biology, Yale University, New Haven, Connecticut, USA; c Howard Hughes Medical Institute, Yale West Campus, West Haven, Connecticut, USA; d Microbiology Program, Yale University, New Haven, Connecticut, USA; e Laboratory of Bacteriology, Rocky Mountain Laboratories, Division of Intramural Research, National Institute of Allergy and Infectious Diseases, National Institutes of Health, Hamilton, Montana, USA; f Department of Microbial Pathogenesis, Yale School of Medicine, New Haven, Connecticut, USA; University of Georgia

**Keywords:** *Borrelia*, *Borrelia burgdorferi*, Lyme disease, LysM, outer membrane, antibiotic, fluorescent protein, image analysis, promoters, spirochetes

## Abstract

Lyme disease is the most widely reported vector-borne disease in the United States. Its incidence is rapidly increasing, and disease symptoms can be debilitating. The need to understand the biology of the disease agent, the spirochete Borrelia burgdorferi, is thus evermore pressing. Despite important advances in B. burgdorferi genetics, the array of molecular tools available for use in this organism remains limited, especially for cell biological studies. Here, we adapt a palette of bright and mostly monomeric fluorescent proteins for versatile use and multicolor imaging in B. burgdorferi. We also characterize two novel antibiotic selection markers and establish the feasibility of their use in conjunction with extant markers. Last, we describe a set of promoters of low and intermediate strengths that allow fine-tuning of gene expression levels. These molecular tools complement and expand current experimental capabilities in B. burgdorferi, which will facilitate future investigation of this important human pathogen. To showcase the usefulness of these reagents, we used them to investigate the subcellular localization of BB0323, a B. burgdorferi lipoprotein essential for survival in the host and vector environments. We show that BB0323 accumulates at the cell poles and future division sites of B. burgdorferi cells, highlighting the complex subcellular organization of this spirochete.

**IMPORTANCE** Genetic manipulation of the Lyme disease spirochete B. burgdorferi remains cumbersome, despite significant progress in the field. The scarcity of molecular reagents available for use in this pathogen has slowed research efforts to study its unusual biology. Of interest, B. burgdorferi displays complex cellular organization features that have yet to be understood. These include an unusual morphology and a highly fragmented genome, both of which are likely to play important roles in the bacterium’s transmission, infectivity, and persistence. Here, we complement and expand the array of molecular tools available for use in B. burgdorferi by generating and characterizing multiple fluorescent proteins, antibiotic selection markers, and promoters of varied strengths. These tools will facilitate investigations in this important human pathogen, as exemplified by the polar and midcell localization of the cell envelope regulator BB0323, which we uncovered using these reagents.

## Abstract (precis)

Genetic manipulation of the Lyme disease spirochete B. burgdorferi remains cumbersome, despite significant progress in the field. The scarcity of molecular reagents available for use in this pathogen has slowed research efforts to study its unusual biology. Of interest, B. burgdorferi displays complex cellular organization features that have yet to be understood. These include an unusual morphology and a highly fragmented genome, both of which are likely to play important roles in the bacterium’s transmission, infectivity, and persistence. Here, we complement and expand the array of molecular tools available for use in B. burgdorferi by generating and characterizing multiple fluorescent proteins, antibiotic selection markers, and promoters of varied strengths. These tools will facilitate investigations in this important human pathogen, as exemplified by the polar and midcell localization of the cell envelope regulator BB0323, which we uncovered using these reagents.

## INTRODUCTION

Lyme disease, a widespread infection transmitted by hard ticks of the Ixodes genus, is the most prevalent vector-borne disease in the United States. The disease is also common in Europe and Asia, and its incidence and geographic distribution have been steadily increasing in recent decades (1). Lyme disease is caused by spirochetes belonging to the Borrelia burgdorferi *sensu lato* group, with B. burgdorferi *sensu stricto* (here referred to as B. burgdorferi) being the principal agent in North America, and Borrelia afzelii and Borrelia garinii being the primary agents in Eurasia. In humans, acute Lyme disease is often associated with a characteristic skin rash and flu-like symptoms. If left untreated, late stages of infection may result in carditis, neurological manifestations, and arthritis (2).

Spirochetes in general, and Borrelia species in particular, display cellular features unusual for bacteria (3). Spirochete cells are typically very long and thin by bacterial standards. B. burgdorferi cells, for example, are 10 to 25 μm long and ∼250 nm wide (4–6). Spirochetes are also highly motile, but, unlike most bacteria, their flagella are not external organelles (7). Instead, these flagella are located in the periplasm (i.e., between the inner and outer membranes). In B. burgdorferi, the helicity of the flagella imparts the flat-wave morphology of the bacterium (8). B. burgdorferi also possesses what is likely the most segmented genome of any bacterium investigated to date. It is made up of a linear chromosome of about 900 kb and over 20 linear and circular genetic elements ranging from 5 to 60 kb in length (9, 10). These smaller genetic elements are often referred to as plasmids, though many of them encode proteins that are essential for the life cycle of this organism (11). Recent work from our laboratory has shown that Borrelia species also have an uncommon pattern of cell wall synthesis in which discrete zones of cell elongation in one generation predetermine the division sites of daughter cells in the next generation (6).

While these unusual cellular features are integral to B. burgdorferi physiology and pathogenesis, little is known about how they arise or are maintained over generations. In fact, the cell biology of this pathogen remains largely unexplored. Technical hurdles have slowed progress in this area. Genetic manipulation of B. burgdorferi is feasible, but the available genetic tools are still limited, and the process remains cumbersome (12, 13). Constitutive gene expression is mostly limited to the use of very strong promoters. Moreover, apart from a few exceptions (14–19), fluorescent protein reporters have primarily been used as gene expression reporters or as cellular stains for *in vivo* localization of the spirochete (13). Yet, fluorescent proteins have many more uses which have transformed the field of cell biology (20). For example, fluorescent proteins have opened the door to localization studies in live cells. They have also facilitated the detection of protein-protein interactions, the measurement of physical properties of cells, and the investigation of single events and of population heterogeneity. Much of this information is not accessible through the use of bulk biochemical measurements on cell populations. The averaging inherent to such techniques leads to a loss of spatial resolution and obscures rare events and cell-to-cell or subcellular heterogeneity of behavior (21). Indeed, the ability to perform extensive genetic manipulations and to use a wide panel of fluorescent proteins in an organism has been key to progress in understanding bacterial cell biology (22). Such approaches have been used extensively in model bacteria, including Bacillus subtilis, Escherichia coli, and Caulobacter crescentus, since the first reported use of fluorescent protein fusions two decades ago (23–25). In order to facilitate the study of B. burgdorferi, we have generated new investigative tools by characterizing a panel of fluorescent proteins, promoters, and antibiotic resistance markers for use in this medically important bacterium. We exemplify the usefulness of these reagents by creating an mCherry fusion to BB0323, a multifunctional B. burgdorferi lipoprotein required for outer membrane stability (26–28) and that is essential for the spirochete’s survival in the tick vector and the mammalian host (27). Using this fusion, we show that BB0323 localizes at the spirochete’s poles and at future division sites, highlighting underappreciated spatial and temporal organization principles of B. burgdorferi cells. (Earlier versions of this article were submitted to the online preprint archive BioRxiv [29]).

## RESULTS

### Wide palette of fluorescent proteins for imaging in B. burgdorferi.

Only a few fluorescent proteins have been used to date in B. burgdorferi (summarized in Table 1). These proteins belong primarily to two color classes, green fluorescent proteins (GFP) and red fluorescent proteins (RFP) (Table 1). To expand the range of options for multicolor imaging of B. burgdorferi, we focused on a set of fluorescent proteins that have been used in localization studies in other organisms and codon-optimized their genes for translation in B. burgdorferi. The selected proteins span five color classes (Table 1), and their signals can be collected using widely available filter sets for cyan fluorescent protein (CFP), GFP, yellow fluorescent protein (YFP), mCherry/TexasRed, and Cy5.5 fluorescence. The selected cyan, green, and yellow variants are all derivatives of the jellyfish (Aequorea victoria) GFP. We used both the classic variants Cerulean (30), enhanced GFP (EGFP) (31), Citrine (32), and the superfolder (e.g., sfGFP) variants (33). All variants included the monomeric mutation A206K (34), denoted by a lowercase “m” before the name of the protein (e.g., mCerulean). Our red protein of choice was mCherry (35), a monomeric improved variant of mRFP1 (36). Last, we codon-optimized and expressed an infrared fluorescent protein (iRFP) (37). The far-red wavelengths used to excite this fluorophore are less toxic to cells than the shorter excitation wavelengths used for the other fluorescent proteins, and the sample autofluorescence in the near-infrared spectral region is lower than in the other blue-shifted imaging windows (20, 38).

**TABLE 1:** Fluorescent proteins used in *B. burgdorferi*

Color class	Protein expressed	Ex/Em max (nm)^*a*^	Source or reference for protein/gene development	Reference for use in B. burgdorferi	Notes
Fluorescent proteins previously used in B. burgdorferi					
Cyan	CFP	434/477^*b*^	Clontech; 107	108	Rarely used
Green	EGFP	489/509	Clontech; 31	62	Low expression, has mammalian codon usage
	GFPmut1	488/507	107, 109–111	108	Widely used, adapted for bacterial expression, same protein as EGFP
	GFPmut3	501/511	109	102	
	GFP cycle 3	NR^*c*^	112	113	Retains UV excitation peak
Yellow	YFP	514/527^*b*^	107	108	Rarely used
Red	mRFP1	584/607	36	18	Folds in the periplasm
	dTomato	554/581	35	114	Dimeric
New fluorescent proteins adapted for use in B. burgdorferi					
Cyan	mCerulean	433/475	30	This study	A206K monomeric mutation
	msfCFP	NR^*c*^	33	This study	A206K mutation, superfolder
Green	mEGFP	489/509	31	This study	A206K mutation
	msfGFP	485/NR^*c*^	33	This study	A206K mutation, superfolder
Yellow	mCitrine	516/529	32	This study	A206K mutation
	msfYFP	NR^*c*^	33	This study	A206K mutation, superfolder
Red	mCherry	587/610	35	This study	
Infrared	iRFP	690/713	37	This study	Dimeric

a Maximum (max) excitation (Ex) and emission (Em) wavelengths.

b Values assumed to be those for ECFP and EYFP, respectively (20).

c NR, not reported. Values were not reported in the original publication or could not be exactly inferred from excitation and emission graphs.

**FIG 1:**
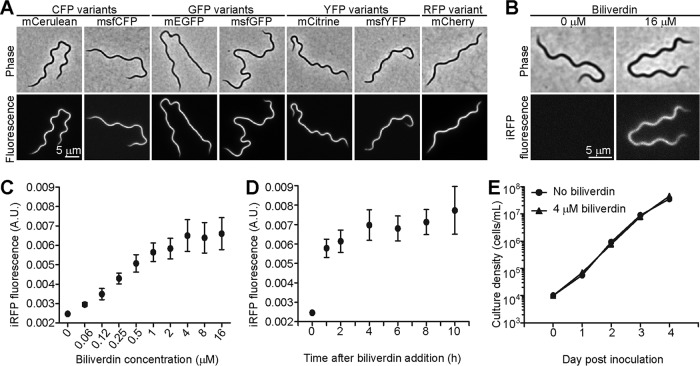
Fluorescent protein characterization. (A) *B. burgdorferi* strains CJW_Bb090 through CJW_Bb096 expressing the indicated fluorescent proteins were imaged with matching filter sets. (B) Strain CJW_Bb100 expressing iRFP requires biliverdin for development of fluorescence. Cells were grown in liquid culture with biliverdin for 2 days prior to imaging using a Cy5.5 filter set. (C) Dose response of iRFP fluorescence to biliverdin concentration. Strain CJW_Bb100 was grown in the presence of biliverdin for 2 days prior to imaging. Between 86 and 206 cells were analyzed for each concentration. Total cellular fluorescence levels were normalized by the cell area. Shown are means ± standard deviations (SD). A.U., arbitrary units. (D) Time course of iRFP fluorescence development in strain CJW_Bb100 following the addition of 16 μM biliverdin. Between 68 and 110 cells were analyzed for each time point. (E) Biliverdin does not affect *B. burgdorferi* growth. Strain CJW_Bb100 was inoculated at 10^4^ cells/ml in duplicate in medium containing 4 μM biliverdin or no biliverdin, after which the spirochetes were enumerated daily.

To visualize these fluorescent proteins, we expressed them in strain B31 e2 from the strong flagellin promoter P*_flaB_* (39) located on a shuttle vector. With the exception of iRFP, each fluorescent protein displayed bright fluorescence when imaged using a filter set matched to its color (Fig. 1A). Unlike the other fluorescent proteins, which oxidatively conjugate their own amino acid side chains to create a fluorophore (20), iRFP covalently binds an exogenous biliverdin molecule, which then serves as the fluorophore (37). Adding the biliverdin cofactor to the growth medium of the iRFP-expressing strain rendered the cells fluorescent in the near-infrared region of the spectrum, as detected with a Cy5.5 filter set (Fig. 1B). Treating a control strain carrying an empty shuttle vector with biliverdin did not cause any increase in cellular fluorescence (data not shown). To measure cellular fluorescence levels, we chose a microscopy-based approach in conjunction with quantitative image analysis. This allowed us to efficiently analyze hundreds of cells and to clearly distinguish individual cells from similarly sized debris found in the culture medium, or from clumps of multiple cells. Using this method, we established that a 4 μM concentration of biliverdin in the growth medium was sufficient to achieve maximal cellular brightness (Fig. 1C). Close-to-maximal iRFP brightness was reached as early as an hour after the addition of biliverdin to the culture and was maintained throughout subsequent growth (Fig. 1D). Furthermore, continuous growth of B. burgdorferi in the presence of biliverdin was indistinguishable from growth in biliverdin-free medium (Fig. 1E). This indicates that culture experiments that involve iRFP may be performed either by adding biliverdin shortly before imaging or by growing the cells continuously in the presence of biliverdin.

**FIG 2:**
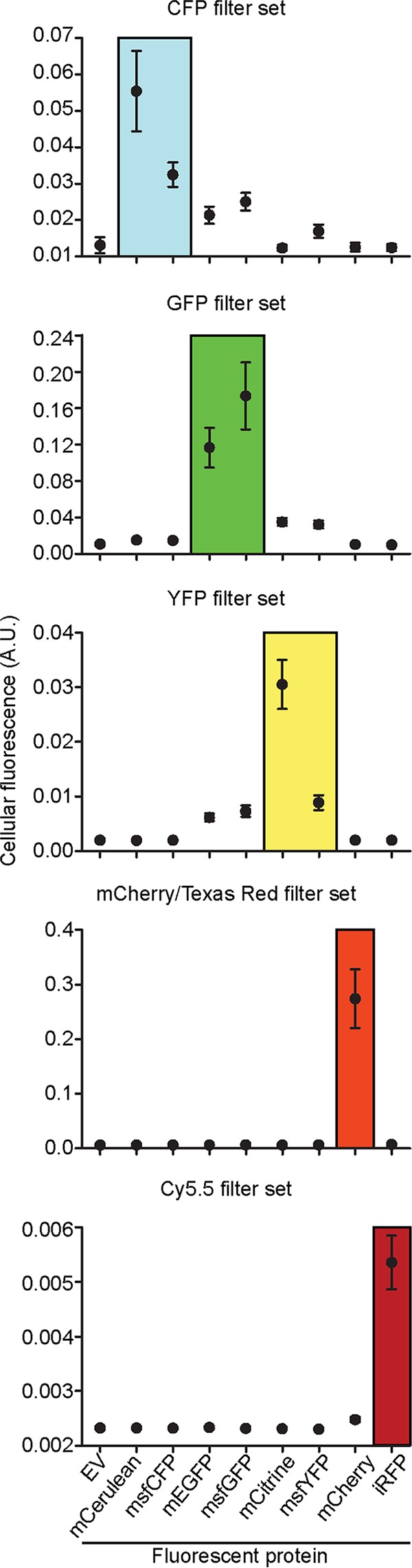
Quantification of fluorescent protein signal using common fluorescence filter sets. Strains CJW_Bb090 through CJW_Bb096 and CJW_Bb100 expressing the fluorescent proteins indicated at the bottom of the figure were each imaged using five filter sets: CFP, GFP, YFP, mCherry/TexasRed, and Cy5.5 (see Materials and Methods for filter set specifications). Strain CJW_Bb073 carrying an empty shuttle vector (EV) was also imaged to measure the cellular autofluorescence. Each filter set is listed at the top of the corresponding graph. Fluorescence intensity values were normalized by the cell area and are depicted as means ± SD in arbitrary units (A.U.). For each strain, 117 to 308 cells were analyzed. The iRFP strain was grown in the presence of 4 μM biliverdin for 3 days prior to imaging. The boxed region in each plot highlights the data obtained with filter sets that were ideal for the expressed fluorescent protein.

In microscopy studies, simultaneous imaging of multiple fluorescent proteins requires that the signal generated by a given fluorescent protein does not bleed into the fluorescence channels used to collect the signal of another protein. To assess the viability of using our palette of fluorescent proteins for multicolor imaging in B. burgdorferi, we quantified the signal generated by each fluorescent protein when imaged with the commonly used CFP, GFP, YFP, mCherry, and Cy5.5 filter cubes (Fig. 2). We found that each fluorescent protein generated a strong signal when imaged with a color-matched filter set (Fig. 2). As expected, we detected a significant spectral overlap between CFP and GFP, as well as between GFP and YFP variants. Importantly, signal quantification showed that mCerulean or msfCFP can be imaged alongside mCitrine, mCherry, and iRFP, while mEGFP or msfGFP can be imaged alongside mCherry and iRFP, opening the door to combinatorial imaging of up to four proteins in the same B. burgdorferi cell.

### Promoters for various levels of expression in B. burgdorferi.

To date, constitutive expression of exogenous genes in B. burgdorferi, including antibiotic selection markers and reporter genes, such as those for fluorescent proteins and luciferases, has almost exclusively relied on very strong promoters, such as P*_flaB_* and P*_flgB_* (13, 39). Reporter expression from strong promoters facilitates spirochete detection, particularly in high-fluorescence-background environments, such as the tick midgut or mammalian tissues (40–42). However, as overexpression can affect protein localization, interfere with function, or cause cellular toxicity (e.g., references 43–53), lower levels of gene expression have proven instrumental in facilitating localization studies (e.g., references 54–57) and are often preferred in such applications.

**FIG 3:**
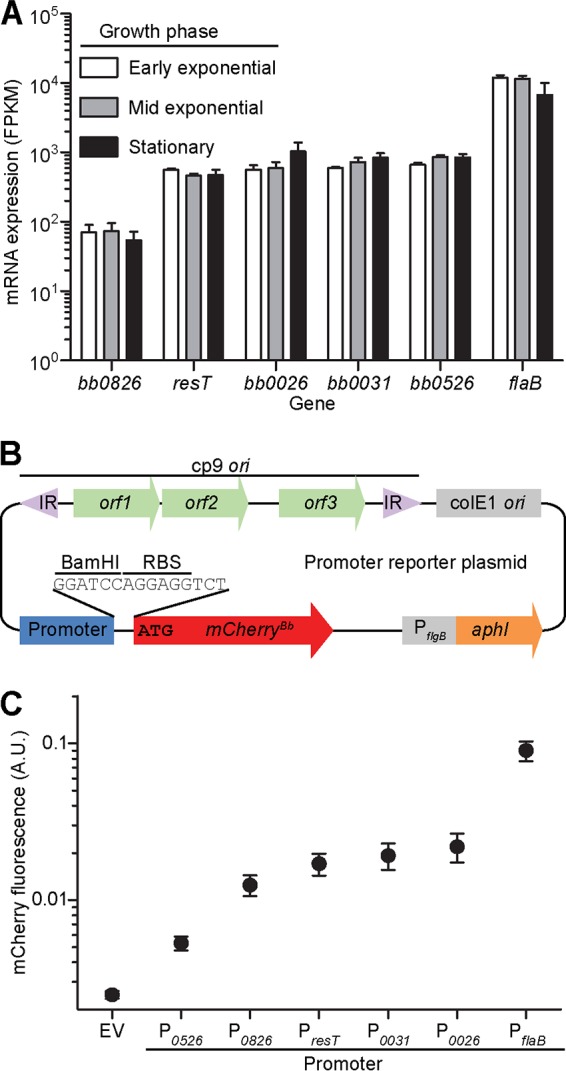
Promoter strength quantification. (A) mRNA expression levels extracted from published RNA-seq data obtained using strain B31-A3 (106) grown to early exponential phase (10^6^ cells/ml), mid-exponential phase (10^7^ cells/ml), or stationary phase (1 day after reaching 10^8^ cells/ml) (58). FPKM, fragments per kilobase transcript per million mapped reads. (B) Promoter reporter plasmid map (not drawn to scale). IR, inverted repeats; cp9 *ori*, origin of replication of *B. burgdorferi* plasmid cp9, which includes the genes *orf1*, *orf2*, and *orf3* needed for plasmid replication in *B. burgdorferi*; colE1 *ori*, *E. coli* origin of replication; P*_flgB_*, *B. burgdorferi* flagellar rod operon promoter; *aphI*, kanamycin resistance gene. The promoter (blue) and the mCherry-coding sequence (red) are connected by a BamHI restriction enzyme site and a ribosomal binding site (RBS). The BamHI-RBS-mCherry sequence effectively replaced the native gene’s protein coding sequence. The translational start site is marked by the ATG codon. (C) Promoter strength quantified by measuring cellular mCherry fluorescence in strains CJW_Bb069, CJW_Bb108 through CJW_Bb112, and CJW_Bb146. The fluorescence levels were normalized by cell area. The promoters were ranked in increasing order of the mean fluorescence values and are listed below the graph. Shown are means ± SD. Between 97 and 160 cells were analyzed per strain. EV, empty vector; A.U., arbitrary units.

To identify promoters of low and medium strengths, we mined a published RNA sequencing (RNA-seq) data set that measured transcript levels in cultures of B. burgdorferi in early exponential, mid-exponential, and stationary phases of growth (58). We selected five genes whose expression was largely unchanged among the three growth phases tested (Fig. 3A), amplified a DNA region upstream of each gene’s predicted translational start site, and fused it to an mCherry reporter in a kanamycin resistance-conferring shuttle vector (Fig. 3B). The amplified putative promoter sequences ranged in size from 129 to 212 bp and included the reported 5′ untranslated regions (5′UTRs) of the downstream genes (58, 59). We also included in our analysis an empty vector and a vector containing a P*_flaB_-mCherry^Bb^* fusion, which served as references for no and high expression, respectively.

We transformed these constructs into B. burgdorferi, imaged the resulting strains, and quantified the fluorescence level in each cell. All promoters elicited fluorescence levels above the background of the strain carrying the empty vector (Fig. 3C). We noticed differences between the RNA-seq and mCherry reporter-based methods of measuring promoter strength, as detailed in the Discussion. Importantly, however, the promoters we tested displayed a broad dynamic range from low (P*_0526_*) to intermediate (P*_0826_*, P*_resT_*, P*_0031_*, and P*_0026_*), to high (P*_flaB_*) strength.

### Antibiotic selection in B. burgdorferi using hygromycin B and blasticidin S resistance markers.

Several antibiotic resistance markers have been used to perform genetic manipulations in B. burgdorferi and have recently been reviewed in detail (13). The most widely used today are the kanamycin (*aphI*), gentamicin (*aacC1*), streptomycin (*aadA*), and erythromycin (*ermC*) resistance genes (see Table 2) (39, 60–62). The use of several other antibiotics for selection is either ineffective (e.g., zeocin, chloramphenicol, and puromycin), discouraged due to safety concerns (e.g., tetracyclines, β-lactams, and sometimes erythromycin), redundant due to cross-resistance (several aminoglycoside antibiotics), or no longer widespread (coumermycin A_1_) due to alterations in cell physiology induced by both the antibiotic and the resistance marker (13, 61).

**TABLE 2:** Summary of antibiotic resistance markers used in *B. burgdorferi*^*a*^

Resistance gene	Antibiotic	MIC in liquid culture (μg/ml)	Notes	Reference or source
Widely used resistance markers				
*aphI*	Kanamycin	<25	Cross-resistance to neomycin, lividomycin, paromomycin, and ribostamycin	39, 61
*aadA*	Streptomycin	7^*b*^	Expected cross-resistance to spectinomycin	60
*aacC1*	Gentamicin	<15.6		61
*ermC*	Erythromycin	0.005	Resistance level varies among strains, may pose safety risk	62, 115, 116
Newly developed resistance markers				
*bsd^*Bb*^*	Blasticidin S	<5	No cross-resistance to the selection antibiotics listed in this table	This study
*hph^*Bb*^*	Hygromycin B	<200	No cross-resistance to the selection antibiotics listed in this table	This study

a For space considerations, this table does not contain a comprehensive list of antibiotic resistance markers developed for use in *B. burgdorferi*. For a detailed discussion of other markers, please see reference 13.

b Value is that of an 50% inhibitory dose (ID_50_).

**FIG 4:**
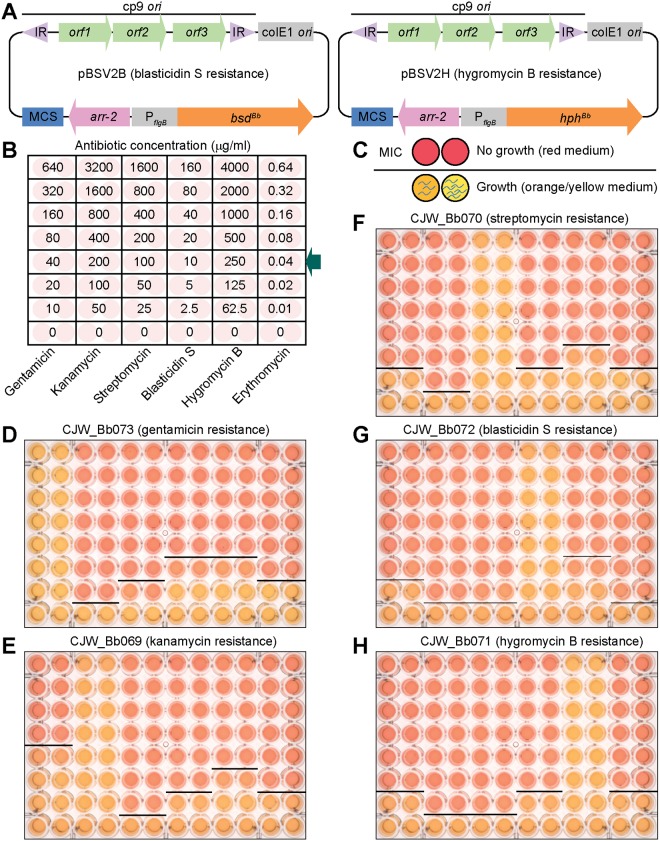
Characterization of blasticidin S and hygromycin B resistances in *B. burgdorferi*. (A) Maps of shuttle vectors pBSV2B and pBSV2H. IR, inverted repeats; cp9 *ori*, origin or replication of *B. burgdorferi* plasmid cp9; colE1 *ori*, *E. coli* origin of replication; MCS, multicloning site; *arr-2*, rifampin resistance gene for selection in *E. coli*; P*_flgB_*, *B. burgdorferi* flagellar rod operon promoter; *bsd^Bb^*, *B. burgdorferi* codon-optimized blasticidin S deaminase-encoding gene; *hph^Bb^*, *B. burgdorferi* codon-optimized hygromycin B phosphotransferase-encoding gene. The maps are not drawn to scale. (B) Plate map showing the final antibiotic concentrations used for cross-resistance testing. Each concentration was tested in two adjacent wells. Concentrations routinely used for selection are indicated by the arrow. (C) Schematic representation of color change of the growth medium from red (absence of spirochete growth) to orange/yellow (presence of spirochete growth). A line marks the boundary between growth and no growth in an antibiotic concentration series. The lowest antibiotic concentration that blocked growth was identified as the MIC. (D to H) Susceptibility test of each resistance-carrying strain to various antibiotic concentrations according to the plate layout shown in panel B. The plates were incubated to allow for growth-dependent acidification of the medium and change in phenol red pH indicator color from red to orange and yellow, as depicted in panel C. Images were obtained using colorimetric imaging of the individual plates. MIC boundaries are marked by dark lines, and the MIC values are summarized in Table 3. The strains used are listed above each image.

To expand the panel of antibiotic resistance markers that can be used in B. burgdorferi, we focused on two antibiotics commonly used for the selection of eukaryotic cells, namely, the translation inhibitors hygromycin B and blasticidin S. Rendering B. burgdorferi resistant to them does not pose a biosafety concern, as these antibiotics are not used to treat Lyme disease (2). We found that hygromycin B and blasticidin S prevented B. burgdorferi growth in liquid culture at concentrations of 200 and 5 μg/ml, respectively (Table 2). For resistance cassettes, we used the E. coli gene *hph* [also known as *aph(4)-Ia*], which encodes a hygromycin B phosphotransferase, and the Aspergillus terreus gene *bsd*, which encodes a blasticidin S deaminase (63–65). We performed codon optimization of these genes for translation in B. burgdorferi and placed them under the control of the strong P*_flgB_* promoter on a shuttle vector (Fig. 4A). The resulting vectors, pBSV2H and pBSV2B, also carry the rifampin resistance gene *arr-2* of Pseudomonas aeruginosa (66–68), which encodes a rifampin ADP-ribosyltransferase. B. burgdorferi is naturally resistant to rifampin (69, 70), but the use of rifampin for selection in E. coli instead of the more expensive blasticidin S and hygromycin B antibiotics reduces the cost of generating and propagating the vectors in E. coli.

B. burgdorferi strains obtained by transforming pBSV2B or pBSV2H into B31 e2 grew readily in cultures containing 10 μg/ml blasticidin S or 250 μg/ml hygromycin B, respectively. Selection with these antibiotics was effective both in liquid culture and in semisolid BSK-agar medium. We used these strains to test whether the antibiotic resistance cassettes encoded by these vectors conferred any cross-resistance to the often-used antibiotics kanamycin, gentamicin, streptomycin, and erythromycin. In parallel, we performed reciprocal tests using B31 e2-derived strains that carried a kanamycin, gentamicin, or streptomycin resistance cassette. Each strain was grown in the presence of 2-fold serial dilutions of each antibiotic (Fig. 4B). Each dilution series was centered on the concentration routinely used for selection with each of the tested antibiotics (Fig. 4B, arrow). We incubated all cultures for at least 4 days and then inspected each well for growth by dark-field imaging. A well was considered to be growth positive if we detected at least one motile spirochete after scanning a minimum of five fields of view. In addition, we further incubated the plates to allow for growth-dependent acidification of the medium. This pH change is easily detected as a change in the color of the medium from red, denoting no growth, to orange or yellow, denoting various degrees of growth (Fig. 4C to H) (61). We confirmed that wells with the lowest antibiotic concentration at which the medium remained red also did not contain motile spirochetes. This concentration was taken to represent the MIC (Fig. 4C, black line). Whenever we exposed a strain to the antibiotic to which it carried a resistance gene, we readily detected growth at all antibiotic concentrations tested (Fig. 4D to H and Table 3), highlighting the efficacy of each resistance marker. Importantly, we did not detect any major cross-resistance between the five resistance markers and the six antibiotics tested (Fig. 4D to H and Table 3). One exception was the kanamycin-resistant strain CJW_Bb069, which was able to grow in the presence of as much as 40 μg/ml gentamicin (Fig. 4E and Table 3), a concentration routinely used for gentamicin selection (61). A slightly larger amount of gentamicin (80 μg/ml) was, however, sufficient to kill this kanamycin-resistant strain (Fig. 4E and Table 3). This low level of cross-resistance may thus necessitate use of a higher dose of gentamicin for selection if the parental strain is already kanamycin resistant.

**TABLE 3:** MIC values related to Fig. 4

Strain (resistance)	MIC of tested antibiotic (μg/ml)					
	Gentamicin	Kanamycin	Streptomycin	Blasticidin S	Hygromycin B	Erythromycin
CJW_Bb073 (gentamicin)	>640	50	50	10	250	0.02
CJW_Bb069 (kanamycin)	80	>3200	25	5	250	0.02
CJW_Bb070 (streptomycin)	20	50	>1600	5	250	0.02
CJW_Bb072 (blasticidin S)	20	50	25	>160	250	0.01
CJW_Bb071 (hygromycin B)	20	50	25	5	>4000	0.02

### Subcellular localization of a B. burgdorferi LysM domain-containing protein.

To highlight the usefulness of our newly generated B. burgdorferi molecular reagents, we fused the gene encoding mCherry to the 3ʹ end of *bb0323* to create a C-terminal fluorescent fusion. The resulting construct was placed under the control of the intermediate-strength promoter P*_0826_*. BB0323 is an important lipoprotein that is required for B. burgdorferi’s natural infection cycle through the tick and the mammalian reservoir (27). This lipoprotein is proteolytically processed into an N-terminal domain mediating outer membrane stability and cell separation and a C-terminal fragment containing a peptidoglycan-binding LysM domain (26, 28, 71). The N- and C-terminal fragments interact with each other and were proposed to help anchor the outer membrane to the peptidoglycan (28).

**FIG 5:**
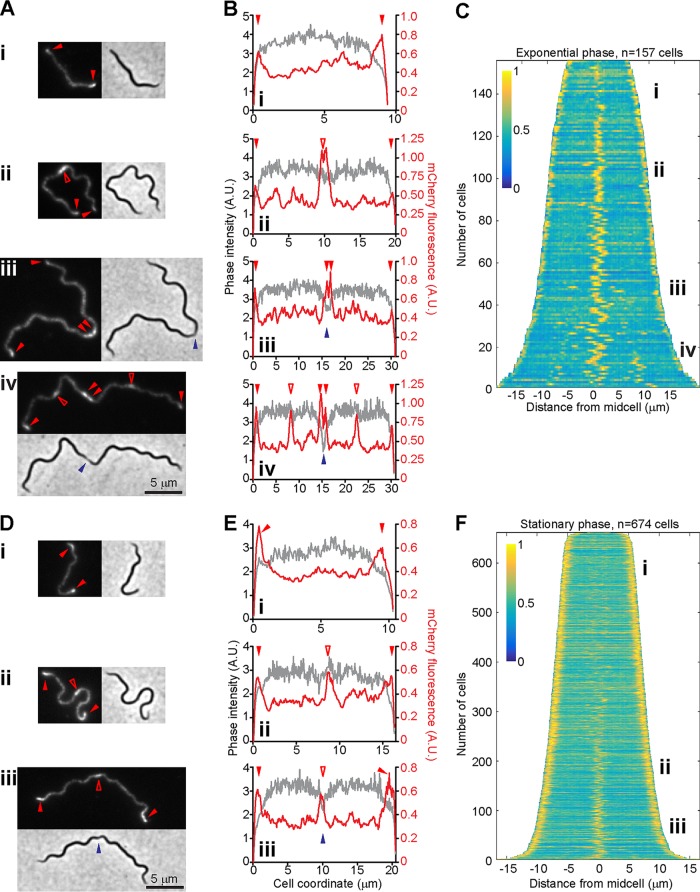
Localization of BB0323 using an mCherry fusion. (A) Micrographs of cells of strain CJW_Bb173 imaged in exponential phase. Shown are mCherry fluorescence and phase-contrast images. All images have the same magnification. Signal accumulation patterns are as follows: i, bipolar; ii, bipolar and midcell in the absence of obvious constriction in the phase-contrast image; iii, bipolar and midcell in the presence of midcell constriction; and iv, bipolar, midcell in the presence of deep cell constriction, and at 1/4 and 3/4 positions along the cell length. (B) Signal quantification along the cell length for cells shown in panel A. The mCherry signal is depicted in red, while the phase-contrast signal is shown in gray. (A and B) Polar localizations and midcell localizations flanking a deep constriction site are marked by filled red arrowheads. Midcell localizations are otherwise marked by empty red arrowheads. Indentation in the phase-contrast signal is marked by blue arrowheads. (C) Demograph depicting the localization of BB0323-mCherry in a population of exponentially growing cells of strain CJW_Bb173. See text for a detailed description. i to iv depict the regions on the demograph where cells with the localization patterns highlighted in panels A and B are located. (D) Micrographs of cells of strain CJW_Bb173 imaged in stationary phase. Shown are mCherry fluorescence and phase-contrast images. All images have the same magnification. Signal accumulation patterns are as follows: i, bipolar; ii, bipolar and midcell in the absence of cell constriction in the phase-contrast image; and iii, bipolar and midcell in the presence of midcell constriction. (E) Signal quantification as a function of cell length for cells shown in panel D. The mCherry signal is depicted in red, while the phase-contrast signal is shown in gray. (D and E) Filled and empty red arrowheads mark polar and midcell localization, respectively, whereas the blue arrowhead shows cell constriction. (F) Demograph depicting the localization of BB0323-mCherry in a stationary-phase population of cells of strain CJW_Bb173. i to iii depict the regions on the demograph where cells with the localization patterns highlighted in panels D and E are located.

We found that the BB0323-mCherry fusion displays striking localization patterns that vary predictably with the culture growth phase (Fig. 5). In an exponentially growing culture, the shortest cells (likely newly born cells) displayed a patchy distribution of the BB0323-mCherry signal along the length of the cell, accompanied by accumulation of this signal at the cell poles (Fig. 5A.i and B.i). In longer cells (i.e., later during the cell cycle), these patchy and bipolar localizations were accompanied by accumulation of the signal at midcell (Fig. 5A.ii and B.ii). These midcell localization events coincided with future division sites (6), though signal accumulation at midcell could be detected in the absence of obvious cell constriction (Fig. 5A.ii and B.ii). Midcell localization persisted through cell constriction, with the fluorescent signal often becoming split into two intensity peaks that flanked the cell constriction site, as shown by the indentation in the phase-contrast signal (Fig. 5A.iii and B.iii). Upon complete cell separation, these pairs of midcell intensity peaks presumably form the polar signals of daughter cells. In a subset of deeply constricted cells, BB0323-mCherry also accumulated at the 1/4 and 3/4 locations along the cell length (Fig. 5A.iv and B.iv), which represent the midcell positions and future division sites of the still-connected daughter cells.

The cell cycle coordination of these localization patterns was confirmed by demograph analysis of static images of an asynchronous population (Fig. 5C). In this population-level representation, each horizontal line represents the distribution of the BB0323-mCherry fluorescent signal along the length of a single cell, as depicted in a heat map. Cells are sorted vertically by their lengths to approximate cell cycle progression. The subsets of the population displaying the types of signal localizations exemplified in Fig. 5A and B are highlighted on the demograph using matching Roman numerals.

In a stationary-phase culture, the same localization patterns were observed, except for the disappearance of the signal at the 1/4 and 3/4 cell positions (Fig. 5D to F). We assume that the lower growth rates in stationary phase ensure that daughter cells fully separate before BB0323-mCherry begins to accumulate at midcell. Lower growth rates in stationary phase may also account for the delayed accumulation of midcell signal during this phase (Fig. 5F) relative to exponential growth (Fig. 5C).

## DISCUSSION

We have undertaken this work to facilitate microscopy-based investigations of the biology of the Lyme disease agent B. burgdorferi. We expanded the available molecular toolkit by characterizing antibiotic resistance markers, fluorescent proteins, and promoters of varied strengths that had not been previously used in this organism.

Alongside the commonly used kanamycin, gentamicin, streptomycin, and erythromycin selection markers, the addition of hygromycin B and blasticidin S resistances as useful selection markers will provide more flexibility in designing genetic modifications. A wider array of non-cross-resistant selection markers is particularly important in the absence of a streamlined method to create unmarked genetic modifications in this bacterium (13). Currently, in infectious B. burgdorferi strains, an antibiotic resistance marker is commonly used to inactivate the restriction modification system encoded by the *bbe02* locus on plasmid lp25. This inactivation increases the efficiency of transformation with shuttle vectors. It also helps maintain this plasmid in the cell population during *in vitro* growth through selective pressure (72–75); this is essential for maintaining a strain’s infectivity, as linear plasmid lp25 is essential *in vivo* but is often rapidly lost during genetic manipulations and growth in culture (76, 77). A second resistance marker is often used to inactivate a gene of interest, either by targeted deletion or by transposon insertion mutagenesis. A third resistance marker is needed for complementation, either at the original locus, or in *trans*. Additional markers are needed if two genes are to be inactivated and complemented simultaneously or if several protein localization reporters need to be expressed both simultaneously and independently.

Today’s cell biology investigations often rely on microscopy studies using fluorescent protein fusions. Prior to our work, green and red fluorescent proteins have been the reporters of choice in B. burgdorferi microscopy studies (Table 1), and only a few subcellular localization and lipoprotein topology studies had been performed using these tools (13–19). We have expanded the palette of fluorescent proteins that can be used in this bacterium by adding several proteins with properties that are highly desirable for imaging and localization studies. These fluorescent proteins are among the brightest of their classes (20, 33, 37), and their spectral properties render them appropriate for simultaneous multicolor imaging of up to four targets. For the most part, they are also monomeric, as all of the A. victoria GFP, CFP, and YFP variants that we have generated carry the A206K mutation (34). Using monomeric fluorescent proteins may be important to prevent artifactual intermolecular interactions, (see, e.g., references 34, 78, and 79). Should the weakly dimeric versions of these proteins be required for specific applications, the A206K mutation can be easily reversed by site-directed mutagenesis. Furthermore, the superfolder variants of these proteins may facilitate tagging when the folding of the fusion protein is otherwise impaired (33). In addition, unlike EGFP, which does not fold in the periplasm of diderm bacteria when exported through the Sec protein translocation system, sfGFP does fold in this compartment (80). It can therefore be an alternative to mRFP1 and mCherry for tagging periplasmic and outer surface-exposed proteins. This is particularly relevant for the study of B. burgdorferi since this bacterium expresses an unusually large number of lipoproteins that are localized on the cell surface or in the periplasmic space (81).

In addition, although dimeric, iRFP may serve as a useful *in vivo* marker and may be preferable to GFP and RFP. Excitation light penetrance in live tissues is better in the far-red/near-infrared region of the spectrum than in the blue-shifted regions used to excite GFP and RFP. Furthermore, tissue autofluorescence in this spectral region is lower, which further facilitates imaging (82, 83). Last, the levels of biliverdin found in animal tissues are in the low-milimolar range, with healthy human plasma containing 0.9 to 6.5 μM biliverdin (84). In our hands, such biliverdin levels are sufficient to elicit maximal fluorescence of B. burgdorferi-expressed iRFP. Furthermore, iRFP has been successfully used to label the bacterium Neisseria meningitidis for *in vivo* imaging (85). Altogether, these considerations suggest that imaging in mice using iRFP-expressing B. burgdorferi should be feasible.

We also characterized promoters of low and intermediate strengths and demonstrated that various degrees of gene expression can be easily achieved in B. burgdorferi. While expression of the genes controlled by these promoters does not change during growth under typical culture conditions (58), we have not ruled out that their expression varies *in vivo*. The relative order of promoter strength, as quantified using the mCherry reporter (Fig. 3C), largely matched the order of the expression levels of the corresponding genes in culture (Fig. 3A) (58), with the exceptions of P*_0526_* and P*_0826_*. While P*_0526_* had an intermediate strength as measured by RNA-seq, it was the weakest when tested using our reporter system. In contrast, P*_0826_* was the weakest promoter based on RNA-seq data but displayed intermediate strength in our experiments. Several factors may cause these differences. For instance, reporter expression from circular shuttle vectors may play a role. The native P*_0526_* and P*_0826_* sequences are located on the chromosome, and differences in DNA topology, including supercoiling, between the chromosome and the circular plasmids are known to affect gene expression in B. burgdorferi (86, 87). Regardless of the reason for these discrepancies, these promoters will facilitate complementation and localization studies where medium and low gene expression levels may be required.

To demonstrate the usefulness of these molecular reagents, we used them to generate and express an mCherry fusion to the LysM domain-containing protein BB0323. LysM domain-containing proteins, including BB0323, have been shown to bind the peptidoglycan layer (28, 88). Strikingly, BB0323-mCherry localized at the cellular poles and at future division sites at midcell throughout most of the cell cycle. Near the end of the cell cycle, before cells separate, BB0323-mCherry also often accumulated at the 1/4 and 3/4 cell positions corresponding to the division sites of future daughter cells. We previously demonstrated that the midcell and the 1/4 and 3/4 positions represent regions of active peptidoglycan synthesis in B. burgdorferi (6). Perhaps BB0323 accumulates at sites of peptidoglycan growth because they differ in chemical composition. A more likely alternative is that these peptidoglycan regions are multilayered. A higher local peptidoglycan concentration would provide a denser binding platform for BB0323, resulting in its accumulation. In fact, cryo-electron tomography imaging of B. burgdorferi has revealed multiple layers of peptidoglycan at division sites (4), as well as a thick peptidoglycan layer at the poles (89). The accumulation of BB0323 at sites of multilayered peptidoglycan would be reminiscent of the LysM-containing protein DipM in the alphaproteobacterium Caulobacter crescentus, which localizes at zones of multilayered peptidoglycan, including division sites and poles, via its LysM domains (90–92). Importantly, the striking cell cycle-coordinated localization of BB0323 demonstrates that proteins with critical functions are spatially distributed in B. burgdorferi, highlighting a layer of regulation that has been poorly explored in spirochetes.

In summary, our study describes novel molecular tools that we hope will aid investigations in the Lyme disease field and spur further progress in the study of this medically important and highly unusual bacterium.

## MATERIALS AND METHODS

### Bacteria, growth conditions, and genetic transformations.

The bacterial strains used in this study are listed in Table 4. E. coli strains were grown at 30°C in liquid culture in Super Broth medium (35 g/liter bacto-tryptone, 20 g/liter yeast extract, 5 g/liter NaCl, 6 mM NaOH) with shaking, or on LB agar plates. Plasmids were transformed by electroporation or heat shock. For the selection of E. coli strains, we used 200 μg/ml (solid medium) or 100 μg/ml (liquid medium) ampicillin, 20 μg/ml (solid medium) or 15 μg/ml (liquid medium) gentamicin, 50 μg/ml kanamycin (solid and liquid media), 50 μg/ml spectinomycin (solid medium), 50 μg/ml streptomycin (liquid medium), and 25 μg/ml (liquid medium), or 50 μg/ml (solid medium) rifampin.

**TABLE 4:** Strains used in this study

Strain	Genotype/description	Antibiotic resistance	Source or reference
E. coli cloning strains			
DH5α	F^-^ Φ80*dlac*ZΔ*M15* Δ(*lacZYA-argF*)*U169 deoR recA1 endA1 hsdR17* (r_k_^−^ m_k_^+^) *phoA supE44* λ^−^ *thi-1 gyrA96 relA1*	None	Promega
XL10-Gold	Tet^r^ Δ(*mcrA*)*183* Δ(*mcrCB-hsdSMR-mrr*)*173 endA1 supE44 thi-1 recA1 gyrA96 relA1 lac* Hte [F′ *proAB lacI*^q^*Z*ΔM15 Tn*10* (Tet^r^) Amy Cam^r^]	Tetracycline, chloramphenicol	Agilent
B. burgdorferi strains			
B31 MI	Low-passage-number derivative of the type strain B31	None	9
B31 e2	Reduced genome noninfectious clone of strain B31	None	117
B31 A34	Reduced genome noninfectious clone of strain B31	None	118
CJW_Bb069	B31 e2/pBSV2_2	Kanamycin	This study
CJW_Bb070	B31 e2/pKFSS1_2	Streptomycin	This study
CJW_Bb071	B31 e2/pBSV2H	Hygromycin B	This study
CJW_Bb072	B31 e2/pBSV2B	Blasticidin S	This study
CJW_Bb073	B31 e2/pBSV2G_2	Gentamicin	This study
CJW_Bb090	B31 e2/pBSV2G_P_flaB_-msfGFP^Bb^	Gentamicin	This study
CJW_Bb091	B31 e2/pBSV2G_P_flaB_-msfCFP^Bb^	Gentamicin	This study
CJW_Bb092	B31 e2/pBSV2G_P_flaB_-msfYFP^Bb^	Gentamicin	This study
CJW_Bb093	B31 e2/pBSV2G_P_flaB_-mCherry^Bb^	Gentamicin	This study
CJW_Bb094	B31 e2/pBSV2G_P_flaB_-mEGFP^Bb^	Gentamicin	This study
CJW_Bb095	B31 e2/pBSV2G_P_flaB_-mCerulean^Bb^	Gentamicin	This study
CJW_Bb096	B31 e2/pBSV2G_P_flaB_-mCitrine^Bb^	Gentamicin	This study
CJW_Bb100	B31 e2/pBSV2G_P_flaB_-iRFP^Bb^	Gentamicin	This study
CJW_Bb108	B31 e2/pBSV2_P_resT_-mCherry^Bb^	Kanamycin	This study
CJW_Bb109	B31 e2/pBSV2_P_0026_-mCherry^Bb^	Kanamycin	This study
CJW_Bb110	B31 e2/pBSV2_P_0031_-mCherry^Bb^	Kanamycin	This study
CJW_Bb111	B31 e2/pBSV2_P_0526_-mCherry^Bb^	Kanamycin	This study
CJW_Bb112	B31 e2/pBSV2_P_0826_-mCherry^Bb^	Kanamycin	This study
CJW_Bb146	B31 e2/pBSV2_P_flaB_-mCherry^Bb^	Kanamycin	This study
CJW_Bb173	B31 A34/pBSV2G_P_0826_-BB0323-mCherry^Bb^	Gentamicin	This study

B. burgdorferi strains were grown in BSK-II medium supplemented with 6% (vol/vol) heat-inactivated rabbit serum (Sigma-Aldrich or Gibco) or in complete BSK-H medium (Sigma-Aldrich), as previously described (93–95). Cultures were incubated at 34°C under 5% CO_2_ atmosphere in a humidified incubator. Antibiotics were used at the following concentrations (unless otherwise indicated): gentamicin at 40 μg/ml, streptomycin at 100 μg/ml, kanamycin at 200 μg/ml, blasticidin S at 10 μg/ml, and hygromycin B at 250 μg/ml. Ampicillin was purchased from Fisher Scientific, blasticidin S and hygromycin B were from Invivogen, and all other antibiotics and biliverdin hydrochloride were from Sigma-Aldrich.

### B. burgdorferi strain generation.

B. burgdorferi electrocompetent cells were prepared as previously described (96, 97) and were transformed with shuttle vector plasmid DNA (usually 30 μg) by electroporation. Electroporated cells were then allowed to recover overnight in BSK-II medium at 34°C. The next day, the transformants were plated in semisolid BSK-agarose medium with appropriate antibiotics, as previously described (96, 97). Individual colonies were then expanded and characterized. Alternatively, antibiotic selection was initiated in liquid medium, and 5-fold serial dilutions of the culture were plated in a 96-well plate (24 wells for each dilution). After 10 to 14 days of incubation, the wells were inspected by microscopy using dark-field illumination. Based on Poisson distribution probability estimated using the Poisson Distribution Calculator hosted at https://stattrek.com/online-calculator/poisson.aspx, when fewer than 20% of the wells of a given dilution were positive for growth, those wells were considered to contain clonal populations, in agreement with a previous report (98). Clones isolated in this manner were further expanded and characterized. When appropriate, fluorescence imaging was used to confirm fluorescent protein expression. Alternatively, selected nonclonal transformant populations were enumerated using C-Chip disposable hemocytometers (INCYTO), using the manufacturer’s instructions, with the following change: counting was done by continuously scanning the full height of the counting chamber for each counting surface to account for the height of the counting chamber being larger than the size of the spirochetes. Enumerated spirochetes were then diluted in BSK-II medium and plated in 96-well plates at an average density of 0.2 cells/well. After 10 to 14 days, clonal growth was confirmed by dark-field microscopy imaging.

### Determination of MICs and antibiotic cross-resistance.

MICs were determined using strain B31 e2 or B31 MI, while cross-resistance testing was done using B31 e2-derived strains that contained shuttle vectors carrying kanamycin, gentamicin, streptomycin, blasticidin S, or hygromycin B resistance markers (see strains CJW_Bb069 through CJW_Bb073 in Table 4). For both tests, antibiotics were 2-fold serially diluted in complete BSK-II or BSK-H medium. For each concentration, 100 μl of antibiotic solution was dispensed into two to four wells of 96-well plates. The cell density of B. burgdorferi cultures was determined by direct counting using dark-field microscopy. The cultures were then diluted to 2 × 10^4^ cells/ml in antibiotic-free medium, and 100 μl of this diluted culture was added to the antibiotic-containing wells to yield an inoculum of 10^4^ cells/ml. The plates were incubated for at least 4 days at 34°C under a 5% CO_2_ atmosphere in a humidified incubator, after which each well was checked for spirochete growth and motility using dark-field microscopy. A well was marked as positive if motile cells were detected. The plates were further incubated for several days, during which bacterial growth-dependent acidification caused the phenol red pH indicator in the medium to change color. This color change was documented using colorimetric transillumination imaging on a GE Amersham imager 600. We verified that growth scoring of each well by dark-field imaging matched the observed medium color change.

### DNA manipulations.

The plasmids used in this study are listed in Table 5. Site-directed mutagenesis was performed using Agilent’s QuikChange Lightning site-directed mutagenesis kit, as per the kit’s instructions. Restriction endonucleases (regular and high-fidelity versions) and ElectroLigase were purchased from New England BioLabs. DNA polymerases were from Thermo Scientific (Platinum PCR supermix), New England BioLabs (Phusion), or TaKaRa (PrimeSTAR). Oligonucleotide primers were synthesized at Integrated DNA Technologies and are listed in Table 6. Gel extraction was performed using the PureLink quick gel extraction kit (Thermo Scientific). DNA minipreps were done using Zyppy plasmid miniprep kit (Zymo Research), while midipreps were done using the Plasmid Plus midi kit (Qiagen) from 50 ml of overnight E. coli cultures in Super Broth. Correct insert DNA sequences were confirmed at Quintarabio or using an in-house Sanger DNA sequencing service at the Yale Keck Biotechnology Resource Laboratory. Codon optimization was performed using the web-based Java Codon Adaptation Tool hosted at www.jcat.de (99) and the codon usage table for B. burgdorferi as stored at www.kazusa.or.jp/codon (100). Codon-optimized DNA sequences were then chemically synthesized at Genewiz. The names of these genes include a *Bb* superscript to indicate that the gene’s nucleotide sequence is codon-optimized for translation in B. burgdorferi (e.g., *iRFP^Bb^*). The name of the protein encoded by such a gene (e.g., iRFP), however, does not include the Bb superscript, as the protein’s amino acid sequence does not differ from that expressed from other versions of the gene.

**TABLE 5:** Plasmids used in this study

Plasmid name	Description	Antibiotic resistance	Reference or source (Addgene ID)
pBSV2G	Gentamicin-resistant B. burgdorferi shuttle vector	Gentamicin	61
pBLS599	pBSV2-derived B. burgdorferi shuttle vector lacking the zeocin resistance gene; expresses *gfpmut3*	Kanamycin	102
pKFSS1	Streptomycin-resistant B. burgdorferi shuttle vector	Streptomycin	60
pMCS-3	Plasmid carrying the rifampin resistance gene *arr-2*	Rifampin	66
pSL1180	Ampicillin-resistant cloning plasmid	Ampicillin	Amersham
pBSV2G_2	Modified gentamicin-resistant B. burgdorferi shuttle vector; has extended multicloning site	Gentamicin	This study (118225)
pBSV2_2	Kanamycin-resistant B. burgdorferi shuttle vector similar to pBSV2 (101); lacks the zeocin resistance gene; has extended multicloning site	Kanamycin	This study (118226)
pKFSS1_2	Modified streptomycin-resistant B. burgdorferi shuttle vector; has extended multicloning site	Streptomycin	This study (118227)
pBSV2B	Blasticidin S-resistant B. burgdorferi shuttle vector; uses rifampin for selection in E. coli	Blasticidin S, rifampin	This study (118228)
pBSV2H	Hygromycin B-resistant B. burgdorferi shuttle vector; uses rifampin for selection in E. coli	Hygromycin B, rifampin	This study (118229)
pBSV2G_P_flaB_-mCherry^Bb^	For expression of mCherry^Bb^ under the control of the strong B. burgdorferi promoter P*_*flaB*_*	Gentamicin	This study (118230)
pBSV2G_P_flaB_-msfGFP^Bb^	For expression of msfGFP^Bb^ under the control of the strong B. burgdorferi promoter P*_*flaB*_*	Gentamicin	This study (118231)
pBSV2G_P_flaB_-msfCFP^Bb^	For expression of msfCFP^Bb^ under the control of the strong B. burgdorferi promoter P*_*flaB*_*	Gentamicin	This study (118232)
pBSV2G_P_flaB_-msfYFP^Bb^	For expression of msfYFP^Bb^ under the control of the strong B. burgdorferi promoter P*_*flaB*_*	Gentamicin	This study (118233)
pBSV2G_P_flaB_-mEGFP^Bb^	For expression of mEGFP^Bb^ under the control of the strong B. burgdorferi promoter P*_*flaB*_*	Gentamicin	This study (118234)
pBSV2G_P_flaB_-mCerulean^Bb^	For expression of mCerulean^Bb^ under the control of the strong B. burgdorferi promoter P*_*flaB*_*	Gentamicin	This study (118235)
pBSV2G_P_flaB_-mCitrine^Bb^	For expression of mCitrine^Bb^ under the control of the strong B. burgdorferi promoter P*_*flaB*_*	Gentamicin	This study (118236)
pBSV2G_P_flaB_-iRFP^Bb^	For expression of iRFP^Bb^ under the control of the strong B. burgdorferi promoter P*_*flaB*_*	Gentamicin	This study (118237)
pBSV2_P_resT_-mCherry^Bb^	For expression of mCherry^Bb^ under the control of the B. burgdorferi promoter P*_*resT*_*	Kanamycin	This study (118238)
pBSV2_P_0026_-mCherry^Bb^	For expression of mCherry^Bb^ under the control of the B. burgdorferi promoter P*_*0026*_*	Kanamycin	This study (118239)
pBSV2_P_0031_-mCherry^Bb^	For expression of mCherry^Bb^ under the control of the B. burgdorferi promoter P*_*0031*_*	Kanamycin	This study (118240)
pBSV2_P_0526_-mCherry^Bb^	For expression of mCherry^Bb^ under the control of the B. burgdorferi promoter P*_*0526*_*	Kanamycin	This study (118241)
pBSV2_P_0826_-mCherry^Bb^	For expression of mCherry^Bb^ under the control of the B. burgdorferi promoter P*_*0826*_*	Kanamycin	This study (118242)
pBSV2_P_flaB_-mCherry^Bb^	For expression of mCherry^Bb^ under the control of the strong B. burgdorferi promoter P*_*flaB*_*	Kanamycin	This study (118243)
pBSV2G_P_0826_-BB0323-mCherry^Bb^	For expression of a BB0323-mCherry fusion under the control of the B. burgdorferi promoter P*_*0826*_*	Gentamicin	This study (118244)

**TABLE 6:** Oligonucleotide primer sequences

Primer name	Sequence (5ʹ to 3ʹ)^*a*^
NT23	ACCGGTCTCGAGGACGTCGCTAGCGGATCCCGGGGTACC
NT24	GATCGGTACCCCGGGATCCGCTAGCGACGTCCTCGAGACCGGTGTAC
NT27	TATA GAGCTCTGTCTGTCGCCTCTTGTGGCTTCC
NT28	CAC GGATCCTCATTCCTCCATGATAAAATTTAAATTTCTGAC
NT100	TAT GGATCCATGGTTAGTAAAGGTGAAGAAG
NT107	CGCG GAGCTCCGAAGTTTATTATTTTATGATTT
NT108	CGC GGATCCATTAATTCAATTATACCAAG
NT109	GTC GAGCTCTATTCTCCATTCTTTTAAAATTATTATCC
NT110	CAC GGATCCTAAGATTACCTTAATATTATACTTAG
NT111	CAG GAGCTCGTTGATATTAAACTTAAAAGCAATATTATTGTTG
NT112	GAC GGATCCAACCTAACCTCAAGAATTAAATAATAC
NT113	TAT GAGCTCCTTGTTTTCAATGATAGGTTTTTTAGG
NT114	TAT GGATCCATGATGATTCTAATCATAAAAAATCAAAATATC
NT115	TAT GAGCTCGGCAATAGAAGAATCTATAGAAAGC
NT116	CAC GGATCCAATTTATTATAAACTTCATTGCTGTTAAC
NT160	CGC AAGCTTATTTATATAATTCATCCATACCATGAGTAATACC
NT161	TAT GGATCCATGAGTAAAGGTGAAGAATTATTTACTGGTG
NT169	CAG CCTAGGTTAATCTTCAATAACATGTAAACCACG
NT170	TAT CGGCCGCATGGCTTGTTATGACTG
NT171	TAT CTGCAGCATATGGCTAAACCTTTAAGTCAAG
NT172	TAT ACGCGTAAGCCGATCTCGGCTTG
NT173	TAT CGGCCGTACCCGAGCTTCAAGGAAG
NT174	GAG CATATGATGGAAACCTCCCTCATTTAAAATTGC
NT187	CTACTAAAACATTGAACACCCCAAGTTAAAGTAGTAACTAAAGTAGGCCAAG
NT188	CTTGGCCTACTTTAGTTACTACTTTAACTTGGGGTGTTCAATGTTTTAGTAG
NT189	TCTTTACTTAATTTACTTTGATAACTTAAATAATGATTATCAGGTAATAAAACAGGACCATCAC
NT190	GTGATGGTCCTGTTTTATTACCTGATAATCATTATTTAAGTTATCAAAGTAAATTAAGTAAAGA
NT193	CAG AAGCTTATTTATATAATTCATCCATACCACCTG
NT342	TAT GGATCCAGGAGGTTCATGGTTAGTAAAGGTGAAGAAGATAATATGG
ZAK51	TGA GTTAACAGCAATGAAGTTTATAATAAATT GGATCCAGGAGGTCTATGAATATAAAGAATAAATTAATATCGCTGC
ZAK52	TGA GGTACCCTTTGGCAGGAATTATTATCTTCCAGTTAGAATG

a Restriction enzyme sites are underlined.

### Expansion of the multicloning site of extant shuttle vectors.

The multicloning site of the shuttle vectors kanamycin-resistant pBSV2 (101), gentamicin-resistant pBSV2G (61), and streptomycin-resistant pKFSS1 (60) was modified to facilitate cloning by including several additional restriction enzyme sites. The modified vectors were named pBSV2_2, pBSV2G_2, and pKFSS1_2, respectively. The multicloning site of the original vectors contains the following restriction enzyme sites, in order: SacI-**KpnI-XmaI-BamHI**-XbaI-SalI-PstI-SphI-HindIII. The expanded multicloning site contains the following restriction enzyme sites, in order: SacI-**AgeI-XhoI-AatII-NheI-BamHI-XmaI-KpnI**-XbaI-SalI-PstI-SphI-HindIII. The regions of the multicloning site that were modified are marked in bold letters. We note that the AatII and XmaI sites are not unique in shuttle vector pKFSS1_2 and that the XhoI site is not unique in shuttle vectors pBSV2_2 and pKFSS1_2. To construct pBSV2G_2, the multicloning site of the shuttle vector was extended by annealing primers NT23 and NT24 and ligating the product into BamHI/KpnI-digested pBSV2G. To construct pBSV2_2, the SacI/BbsI fragment of pBSV2G_2 containing the extended multicloning site and part of the flagellar rod operon promoter (P*_flgB_*) was cloned into the SacI/BbsI sites of pBLS599. During derivation of pBLS599 from pBSV2, the zeocin cassette of pBSV2 was removed (102). Thus, pBSV2_2 differs from pBSV2 in that it lacks the zeocin resistance cassette and has an expanded multicloning site. To construct pKFSS1_2, the BbsI/SacI fragment of pBSV2G_2 was moved into the BbsI/SacI sites of pKFSS1.

### New shuttle vectors carrying blasticidin S and hygromycin B antibiotic resistance markers.

To construct pBSV2B, the following three fragments were assembled in order into the cloning plasmid pSL1180: (i) the *arr-2* rifampin resistance gene, including its promoter, was PCR amplified from plasmid pMCS-3 using primers NT169 and NT170, digested with AvrII and EagI, and inserted into the AvrII/EagI sites of pSL1180 to form pSL1180_arr2; (ii) a B. burgdorferi codon-optimized blasticidin S deaminase gene, *bsd^Bb^*, was synthesized. It was then PCR amplified with NT171 and NT172, digested with PstI and MluI, and inserted into the PstI/MluI sites of pSL1880_arr2 to form pSL1180_arr2-bsd^Bb^, (iii) the P*_flgB_* sequence of pBSV2G was amplified using NT173 and NT174, digested with EagI and NdeI, and inserted into the EagI/NdeI sites of pSL1180_arr2-bsd^Bb^ to yield pSL1180_arr2-P_flgB_-bsd^Bb^. The resulting *arr2*-P*_flgB_-bsd^Bb^* cassette was excised using MluI and AvrII and ligated into the MluI/AvrII backbone of pBSV2G_2. To construct pBSV2H, a B. burgdorferi codon-optimized hygromycin B resistance gene, *hph^Bb^*, was synthesized. This gene was moved as an NdeI/MluI fragment into the NdeI/MluI backbone of pSL1180_arr2-P_flgB_-bsd^Bb^, an intermediate for the construction of pBSV2B (see above), thereby yielding pSL1180_arr2-P_flgB_-hph^Bb^. The resulting *arr2*-P*_flgB_-hph^Bb^* cassette was excised using MluI and AvrII and ligated into the MluI/AvrII backbone of pBSV2G_2.

### Constructs for expression of fluorescent proteins from the flagellin promoter.

B. burgdorferi codon-optimized fluorescent protein-coding genes *mCerulean^Bb^*, *mEGFP^Bb^*, *msfGFP^Bb^*, *mCitrine^Bb^*, *mCherry^Bb^*, and *iRFP^Bb^* were synthesized. Site-directed mutagenesis was performed on the *msfGFP^Bb^* sequence using the NT187/NT188 and NT189/N190 primer pairs to introduce the Y66W and T203Y mutations and create the *msfCFP^Bb^* and *msfYFP^Bb^* genes, respectively. A BamHI (GGATCC) site was included immediately upstream of the Start ATG codon either during gene synthesis or during PCR amplification of the fluorescent protein-encoding genes. A HindIII site was included, overlapping and downstream of the stop TAA codon (as a TAAGCTT sequence, with the HindIII site underlined), either during gene synthesis or during PCR amplification. *mCherry^Bb^* was PCR amplified using NT100 and NT193. *msfCFP^Bb^* and *msfYFP^Bb^* were PCR amplified using NT160 and NT161. The BamHI/HindIII site-flanked fluorescent protein-encoding genes were released from PCR products or parental plasmids using BamHI and HindIII. The flagellin promoter (P*_flaB_*) sequence (39) was PCR amplified from B. burgdorferi genomic DNA using primers NT27 and NT28 and digested with SacI and BamHI. For each transcriptional fusion to P*_flaB_*, a P*_flaB_* SacI/BamHI fragment and a BamHI/HindIII fragment of the fluorescent protein-encoding gene were assembled, via intermediary constructs, into the SacI/HindIII sites of pBSV2G_2 or pBSV2_2, thus yielding pBSV2G_P_flaB_-mCerulean^Bb^, pBSV2G_P_flaB_-msfCFP^Bb^, pBSV2G_P_flaB_-mEGFP^Bb^, pBSV2G_P_flaB_-msfGFP^Bb^, pBSV2G_P_flaB_-mCitrine^Bb^, pBSV2G_P_flaB_-msfYFP^Bb^, pBSV2G_P_flaB_-mCherry^Bb^, pBSV2G_P_flaB_-iRFP^Bb^, and pBSV2_P_flaB_-mCherry^Bb^.

### Promoters for mCherry^Bb^ reporter expression.

Through intermediary constructs, promoter sequences were inserted between the SacI and BamHI sites of pBSV2_2, while the *mCherry^Bb^* gene was amplified using NT193 and NT342, digested using BamHI and HindIII, and inserted into the BamHI/HindIII sites of the same pBSV2_2 backbone, resulting in kanamycin-resistant shuttle vectors carrying *mCherry^Bb^* transcriptional fusions. The primer NT342 contains a ribosome binding site (RBS) sequence (AGGAGG) downstream of the BamHI site (GGATCC) and upstream of the ATG start codon of the mCherry-encoding gene. The full sequence is ggatccAGGAGGctcATG, with the BamHI site and a 3-nucleotide spacer sequence in lowercase letters and the ribosomal binding site (RBS) and the start codon in uppercase letters. The following primers and B31 genomic DNA were used to amplify the various promoters used: NT107 and NT108 (to amplify nucleotides 2187 to 2371 of the reverse strand of the B31 cp26 plasmid; GenBank accession number NC_001903) for the telomere resolvase promoter P*_resT_*, NT109 and NT110 (to amplify nucleotides 25623 to 25751 of the reverse strand of the B31 chromosome; GenBank accession number NC_001318.1) for P*_0026_*, NT111 and NT112 (to amplify nucleotides 29472 to 29669 of the reverse strand of the chromosome) for P*_0031_*, NT113 and NT114 (to amplify nucleotides 535523 to 535703 of the forward strand of the chromosome) for P*_0526_*, and NT115 and NT116 (to amplify nucleotides 870024 to 870235 of the reverse strand of the chromosome) for P*_0826_*. The following constructs were thus obtained: pBSV2_P_resT_-mCherry^Bb^, pBSV2_P_0026_-mCherry^Bb^, pBSV2_P_0031_-mCherry^Bb^, pBSV2_P_0526_-mCherry^Bb^, and pBSV2_P_0826_-mCherry^Bb^.

### pBSV2G_P_0826_-BB0323-mCherry^Bb^.

Through intermediary constructs, the following DNA segments were assembled in the pBSV2G_2 shuttle vector. P*_0826_* was PCR amplified using NT115 and NT116 and inserted as a SacI/BamHI fragment. *mCherry^Bb^* was synthesized with flanking PstI and HindIII restriction endonuclease sites and was transferred into pBSV2G_2 at its PstI and HindIII restriction sites. Last, *bb0323* was PCR amplified using primers ZAK51 and ZAK52, digested with HpaI and KpnI, and inserted into the same sites of the vector. Please note that HpaI is internal to P*_0826_*, but primer ZAK51 contains the P*_0826_* sequence located between the HpaI and BamHI sites (both underlined in the primer sequence provided in Table 6). Thus, the sequence of P*_0826_* is maintained in the final vector.

### Microscopy.

Visualization and counting of live spirochetes were done using a Nikon Eclipse E600 microscope equipped with dark-field illumination optics and a Nikon ×40 0.55 numerical aperture (NA) phase-contrast air objective. Phase-contrast and fluorescence imaging was done on a Nikon Eclipse Ti microscope equipped with a ×100 Plan Apo 1.40 NA phase-contrast oil objective, a Hamamatsu Orca-Flash4.0 V2 digital complementary metal-oxide semiconductor (CMOS) camera, and a Sola light engine (Lumencor), and was controlled by the Metamorph software (Molecular Devices). Alternatively, light microscopy was performed on a Nikon Ti microscope equipped with a ×100 Plan Apo 1.45 NA phase-contrast oil objective, a Hamamatsu Orca-Flash4.0 V2 CMOS camera, and a Spectra X light engine (Lumencor), and was controlled by the Nikon Elements software. Excitation of iRFP was achieved using the 640/30 nm band of the SpectraX system, but higher excitation efficiency (thus, increased brightness) could in theory be obtained using a red-shifted excitation source between 660 and 680 nm. The following Chroma filter sets were used to acquire fluorescence images: CFP, excitation ET436/20x, dichroic T455lp, emission ET480/40m; GFP, excitation ET470/40x, dichroic T495lpxr, emission ET525/50m; YFP, excitation ET500/20x, dichroic T515lp, emission ET535/30m; mCherry/TexasRed, excitation ET560/40x, dichroic T585lpxr, emission ET630/75m; and Cy5.5, excitation ET650/45x, dichroic T685lpxr, emission ET720/60m. For imaging, cultures were inoculated at densities between 10^3^ and 10^5^ cells/ml and grown for 2 to 3 days to reach densities between 10^6^ and 3 × 10^7^ cells/ml. The cells were then immobilized on a 2% agarose pad (6, 103) made with phosphate-buffered saline covered with a no. 1.5 coverslip, after which the cells were immediately imaged live. Images were processed using the Metamorph software. Figures were generated using the Adobe Illustrator software.

### Image analysis.

Cell outlines were generated using phase-contrast images and the open-source image analysis software Oufti (104). Outlines were checked visually for each cell and were extended manually to the full length of the cells when appropriate. When not assigned to single cells or assigned to noncellular debris, outlines were manually removed. The remaining outlines were further refined using the Refine All function of the software. To quantify fluorescence signals, individual cytoplasmic cylinders connected by an outer membrane bridge (i.e., late predivisional cells with two separated cytoplasms) were treated as independent cellular units. For demograph analysis of strain CJW_Bb173, late predivisional cells were considered to form one cell. Fluorescence signal data were added to the cells, and demographs were generated in Oufti. The resulting cell lists were processed using the MATLAB script addMeshtoCellList.m. This script uses the function getextradata.m, which was previously described (104). Single-cell fluorescence intensity values were calculated by dividing the total fluorescence signal inside a cell outline by the cell’s area using the MATLAB-based function CalculateFluorPerCell.m. Final fluorescence data were plotted using the GraphPad Prism 5 software. The number of cells analyzed for each condition is provided in the figure legends.

### Data availability.

Plasmids generated in this study (and their sequences) are available from Addgene or upon request. The DNA sequences of the various genes that were codon optimized for expression in B. burgdorferi have been deposited at GenBank. The MATLAB code used to process cell fluorescence data can be downloaded from GitHub (105).

DNA sequences of codon-optimized genes have been deposited at GenBank under accession numbers MH644044 through MH644053. Plasmids and sequences have been deposited at Addgene under accession numbers 118225 through 118244. MATLAB code, including dependencies, is provided at GitHub under https://github.com/JacobsWagnerLab/published.
